# The value of ovarian hyperstimulation syndrome in predicting pregnancy outcome in women with polycystic ovarian syndrome and candidate for in vitro fertilization: A case-control study

**DOI:** 10.18502/ijrm.v21i11.14655

**Published:** 2023-12-19

**Authors:** Shamim Pilehvari, Nahid Radnia, Somayeh Ahmadiani, Elaheh Talebi-Ghane, Neda Alimohammadi, Zahra Mousaei Tokaldani

**Affiliations:** ^1^Clinical Research Development Unit of Fatemieh Hospital, Department of Gynecology, Hamadan University of Medical Sciences, Hamadan, Iran.; ^2^Modeling of Non-Communicable Diseases Research Center, Hamadan University of Medical Sciences, Hamadan, Iran.; ^3^Clinical Research Development Unit of Fatemieh Hospital, Hamadan University of Medical Sciences, Hamadan, Iran.

**Keywords:** Ovarian hyperstimulation syndrome, Fertilization, Polycystic ovary syndrome.

## Abstract

**Background:** Ovarian hyperstimulation syndrome (OHSS) as a known complication in women with polycystic ovarian syndrome (PCOS) may occur following inducible fertility treatments such as in vitro fertilization (IVF) and can affect the sequels of these treatments. **Objective:** This study aimed to assess the effects of OHSS on pregnancy outcomes through IVF in women with PCOS. Also, we assessed the value of baseline sexual hormones to predict the pregnancy's success. **Materials and Methods:** This case-control study was conducted on 180 consecutive women suffering from PCOS who were candidates for IVF at Fatemieh hospital in Hamadan, Iran, from May-July 2022. The women were assigned to the case group (with OHSS, n = 129) and the control group (without OHSS, n = 51). Measuring the sexual hormones was performed using the enzyme-linked immunosorbent technique. **Results:** In the multivariable logistic regression model, OHSS could not predict the likelihood of clinical or chemical pregnancy following IVF. None of the baseline sexual hormones could predict the successful chemical or clinical pregnancy in PCOS women following IVF. **Conclusion:** OHSS may not influence IVF-related outcomes in PCOS women. The values of sexual hormones may not also determine the pointed outcome.

## 1. Introduction

Polycystic ovarian syndrome (PCOS) is one of the most common disorders of the endocrine system in women of childbearing age, with prevalence ranging from 5-15% in various studies and is one of the most important causes of infertility in women (1). Women with this syndrome include a large group with non-ovulatory problems in the clinic (70-80%) (2). There is still no consensus on definitive diagnostic criteria for PCOS, and due to the wide variety of signs and symptoms of this syndrome, there are challenges in its management and care, especially for fertility recovery. Moreover, the etiology of PCOS is still unclear. However, genetic predisposition, increased insulin secretion, insulin resistance, increased body mass index (BMI), and chemical and environmental contaminants are possible causes of disease (3, 4). Pregnant women with PCOS are at risk for gestational diabetes, preeclampsia, premature birth, increased risk of infant death, and the need for neonatal intensive care units (5, 6). Pregnant women with PCOS may experience different risky conditions during pregnancy, including abortion and congenital malformations in the first trimester, impaired glucose tolerance, gestational diabetes, hypertension, and preeclampsia in the second and third trimesters (7, 8). Due to reproductive complexity in these women caused by hormonal disturbances, in vitro fertilization (IVF) in women with PCOS is associated with various challenges, from poor ovarian response to uncontrolled ovarian response.

Ovarian hyperstimulation syndrome (OHSS) is a complication sourced by excessive ovulation stimulation that occurs in 1-10% of IVF and embryo transfer cycles (9). Although mild OHSS has no clinical significance, severe OHSS is a life-threatening complication (10). Severe cases of OHSS are characterized by severe ascites, marked increases in ovarian size, hydrothorax, oliguria, increased hemoglobin, and electrolyte disturbances (11). In women with PCOS, ovarian stimulation should be programmed according to the serum level of anti-Mullerian hormone (AMH), number of antral follicles, and especially serum follicle-stimulating hormone (FSH) and luteinizing hormone (LH) levels (12). The dose of stimulant gonadotropin should be programmed to achieve an optimal response during a gonadotropin-releasing hormone (GnRH) antagonist cycle. AMH is reported to be a reliable indicator of the ovarian response to controlled ovarian stimulation (13). Overall, the most important risk for PCOS women candidates for IVF is OHSS, which may be predicted by measuring hormones before treatment (14).

This study aimed to first assess the effects of OHSS on pregnancy outcomes related to IVF in PCOS women and second to compare the values of AMH and LH to FSH ratio in women with PCOS treated with IVF with and without OHSS.

## 2. Materials and Methods

This retrospective case-control study was conducted on 180 consecutive women suffering from PCOS who were candidates for IVF at Fatemieh hospital in Hamadan, Iran, from May-July 2022. The inclusion criteria were the diagnosis of PCOS according to Rotterdam criteria scheduling IVF along with the presence or absence of OHSS and were aged between 18 and 42 yr. In this study, the diagnosis of PCOS was based on the Rotterdam criteria (15). Severe systemic diseases such as liver, cardiovascular, and kidney disease or serum testosterone levels > 7 ng/ml, and lack of access to information were excluded from the study (5 people). Patients with serum testosterone values > 7 ng/ml were not included due to their high risk for virilization or severe hyperandrogenemia that could be confounded with the study outcomes (16).

The diagnosis of OHSS was also based on criteria published in 2016 that emphasized moderate abdominal pain, nausea and vomiting, ascites, and bilateral ovarian enlargement (17). PCOS candidates, for IVF, who underwent controlled ovarian stimulation due to the standard cycle based on the diagnosis of OHSS were divided into case and control groups. The case group consisted of people in whom the stimulation of controlled ovulation led to OHSS, while in the control group, the pointed condition was not observed. Overall, 129 women in the OHSS group and 51 in the control group without any evidence of OHSS were finalized for analysis. It should be noted that these 2 groups were age matched. Frozen embryos were transferred in both groups. Embryos were cultured in vitro after Intracytoplasmic sperm injection for 3-5 days. The quality of embryo was evaluated before the transfer approximately 72 hr (8-cell stage) after insemination with a maximum of 3 embryos. Corpus luteal support was provided on the day of oocyte retrieval, with progesterone injections (intramuscular, 50-100 mg/day), until the pregnancy test. If the evidence of OHSS occurrence or the probability of OHSS risk (number of oocytes retrieved > 25, or estradiol > 4800 ng/ml on human chorionic gonadotropin day) was observed, ET would be postponed and good-quality embryos would be cryopreserved (18). Frozen embryo transfer was performed by first administering in the middle of the luteal phase (day 21 of the cycle) gonadotropin-releasing hormone agonist until the beginning of the period or menses. To confirm ovarian suppression, we assessed the baseline hormonal and transvaginal ultrasound. If the ovarian suppression was confirmed, oral estradiol with a dose of 4-6 mg daily was initiated for 7 days. After 10 days, ultrasound was performed and in the condition that the thickness of endometrial was at least 7 mm, trilaminar morphology and estradiol levels were at least 200 pg/mL the embryo transfer scheduled (19). Information about the treatment method, the amount of drugs used, age, BMI, cause of infertility, occurrence, and severity of OHSS, and the serum levels of AMH, FSH, LH, prolactin, estrogen, and both chemical and pregnancy rates were extracted from women's records in hospital and analyzed. The values of serum hormones were measured using commercial enzyme-linked immunosorbent Kits.

### Ethical considerations

The Ethical Committee of Hamadan University of Medical Sciences, Hamadan, Iran approved the study protocol (Code: IR.UMSHA.REC.1401.016). Written informed consent was obtained from all study participants to begin the study.

### Statistical analysis

The descriptive statistics such as mean ± standard deviation and frequency (percentage) were presented for quantitative and categorical variables, respectively. To compare the quantitative variables in 2 groups, provided the normality hypothesis, the *t* test was used, otherwise, the Mann-Whitney test was used. The receiver operating characteristic (ROC) curve analysis was used to assess the value of baseline serum biomarkers to predict pregnancy outcomes. In this regard, the area under the ROC curves higher than 0.75 indicates a significant value of each serum hormone in predicting outcomes of pregnancy. All analysis was applied in SPSS version 23.0 for Windows (IBM, Armonk, New York) and the significant level was considered < 0.05.

## 3. Results

The baseline characteristics of study participants are summarized in table I. On comparison of the 2 groups, no difference was observed in the average age or history of IVF. However, those with OHSS had significantly lower BMI, had a higher number of oocytes, and suffered more from hirsutism. Concerning hormonal status, the mean serum level of AMH was significantly higher in the group with OHSS. At the same time, we found no difference in the levels of prolactin, thyroid stimulating hormone (TSH), FSH, or LH between the 2 groups.

**Figure 1:**
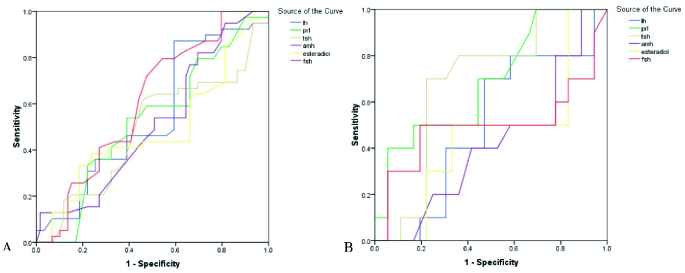
The ROC curve analysis assesses the value of laboratory parameters in predicting the successfulness of chemical pregnancy (A: OHSS [+], B: OHSS [-]).

**Figure 2:**
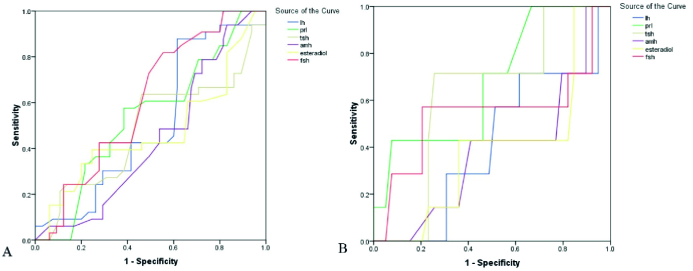
The ROC curve analysis assesses the value of laboratory parameters in predicting the successfulness of clinical pregnancy (A: OHSS [+], B: OHSS [-]).

Regarding IVF and pregnancy outcome, no difference was observed between the 2 groups with and without OHSS in the mean of endometrial thickness (9.17 ± 0.85 vs. 9.21 ± 0.87, p = 0.785) and also the number of embryos transferred (2.98 ± 1.31 vs. 3.04 ± 1.47, p = 0.807). The rate of chemical pregnancy (37.2% vs. 19.6%, p = 0.023) and clinical pregnancy (31.8% vs. 13.7%, p = 0.014) were significantly higher in those with OHSS than in the control group. Still, no difference was found in the rate of abortion between the 2 groups (9.3% vs. 7.8%, p = 0.757). In multivariable logistic regression models (Table II), OHSS could not predict the likelihood of clinical or chemical pregnancy following IVF adjusted for baseline parameters. According to the ROC curve analysis (Table III); none of the baseline sexual hormones could predict the successful chemical or clinical pregnancy in PCOS women with and without OHSS following IVF. In this regard, and as shown in figures 1 and 2, the area under the ROC curves for all hormonal assessments were lower than 0.75 indicating that these factors did not play a role in predicting the outcome of pregnancy.

**Table 1:** Baseline characteristics in the study population


**Characteristics **	**With OHSS (n = 129)**	**Without OHSS (n = 51)**	**P-value**
Age (yr)*	31.16 ± 5.54	30.20 ± 4.81	0.28
BMI (kg/m^2^)*	25.81 ± 3.24	27.23 ± 4.04	0.02
Number of oocytes*	17.80 ± 8.40	13.59 ± 7.67	< 0.001
LH (ng/ml)*	8.03 ± 5.21	8.22 ± 6.46	0.83
Prolactin (mg/dl)*	26.08 ± 2.59	28.36 ± 5.81	0.67
TSH (mg/dl)*	2.28 ± 0.11	2.60 ± 0.19	0.15
AMH (ng/ml)*	8.59 ± 5.25	6.50 ± 4.81	0.01
Stradiol (ng/ml)*	40.31 ± 4.00	47.67 ± 7.83	0.25
FSH (ng/ml)*	5.65 ± 1.92	6.00 ± 1.81	0.28
Duration of PCOS (month)*	5.45 ± 3.32	4.67 ± 2.90	0.14
Irregular menstrual cycle**	61 (47.3)	19 (37.3)	0.22
History of IVF**	42 (33.1)	20 (39.2)	0.43
Amenorrhea**	17 (13.2)	2 (3.9)	0.10
Hirsutism**	45 (34.9)	10 (19.6)	0.04
*Data presented as Mean ± SD. *t* test, **Data presented as number (%). Mann-Whitney test. OHSS: Ovarian hyperstimulation syndrome, BMI: Body mass index, LH: Luteinizing hormone, TSH: Thyroid stimulating hormone, AMH: Anti-Mullerian hormone, FSH: Follicle-stimulating hormone, PCOS: Polycystic ovarian syndrome, IVF: In vitro fertilization			

**Table 2:** The association between OHSS with chemical and clinical pregnancy


	**Chemical pregnancy**			**Clinical pregnancy**		
**Characteristics**	**Beta**	**OR (95% CI)**	**P-value**	**Beta**	**OR (95% CI)**	**P-value**
	**Presence of OHSS**	0.216	1.241 (0.410-3.754)	0.70	-0.207	0.813 (0.219-3.013)	0.75
**Age**	0.031	1.031 (0.931-1.142)	0.55	0.056	1.058 (0.937-1.195)	0.36
**BMI**	0.074	1.077 (0.945-1.227)	0.26	0.233	1.262 (1.066-1.495)	< 0.001
**Number of oocytes **	-0.025	0.975 (0.922-1.031)	0.37	-0.084	0.919 (0.859-0.984)	0.01
**LH**	0.008	1.008 (0.917-1.108)	0.87	0.020	1.021 (0.912-1.142)	0.72
**Prolactin**	-0.006	0.994 (0.980-1.008)	0.37	-0.014	0.986 (0.971-1.002)	0.08
**TSH**	0.134	1.143 (0.776-1.686)	0.49	0.261	1.298 (0.825-2.044)	0.25
**AMH**	-0.004	0.996 (0.910-1.090)	0.93	0.064	1.066 (0.947-1.199)	0.28
**Stradiol**	0.001	1.001 (0.990-1.012)	0.80	0.000	1.000 (0.988-1.012)	0.98
**FSH**	-0.207	0.813 (0.642-1.030)	0.08	-0.402	0.669 (0.495-0.904)	0.00
**Duration of PCOS**	-0.019	0.981 (0.826-1.165)	0.82	-0.024	0.976 (0.796-1.198)	0.81
**Primary type of infertility **	-0.786	0.456 (0.168-1.234)	0.12	-0.662	0.516 (0.162-1.647)	0.26
**Irregular menstrual cycle**	-0.363	0.696 (0.288-1.679)	0.42	-0.214	0.807 (0.298-2.187)	0.67
**History of IVF**	0.671	1.957 (0.789-4.854)	0.14	0.933	2.542 (0.846-7.637)	0.09
**Amenorrhea**	1.902	6.696 (1.360-32.977)	0.01	3.505	33.288 (4.366-53.772)	< 0.001
**Hirsutism **	-0.196	0.822 (0.266-2.540)	0.73	-0.367	0.693 (0.183-2.630)	0.59
The multivariate linear regression analysis. CI: Confidence interval, OR: Odds ratio, OHSS: Ovarian hyperstimulation syndrome, BMI: Body mass index, LH: Luteinizing hormone, TSH: Thyroid-stimulating hormone, AMH: Anti-Mullerian hormone, FSH: Follicle-stimulating hormone, PCOS: Polycystic ovarian syndrome, IVF: In vitro fertilization						

**Table 3:** The receiver operating characteristic curve analysis results to determine the value of baseline hormones for predicting successful clinical and chemical pregnancy


		**With OHSS**			**Without OHSS**		
**Group**		**AUC**	**Standard error**	**P-value**	**AUC**	**Standard error**	**P-value**
**Clinical pregnancy**							
	**LH**	0.545	0.173	0.75	0.410	0.109	0.45
	**Prolactin**	0.673	0.124	0.23	0.674	0.107	0.14
	**TSH**	0.481	0.165	0.89	0.626	0.102	0.29
	**AMH**	0.571	0.138	0.63	0.381	0.112	$0.32^{\#}$
	**Estradiol **	0.276	0.152	0.12	0.386	0.113	0.34
	**FSH**	0.532	0.135	0.82	0.542	0.148	0.72
	**LH/FSH **	0.545	0.173	0.75	0.399	0.089	0.40
**Chemical pregnancy**							
	**LH**	0.643	0.164	0.31	0.500	0.098	0.99
	**Prolactin**	0.714	0.120	0.12	0.688	0.094	0.07
	**TSH**	0.524	0.156	0.86	0.683	0.086	0.07
	**AMH**	0.667	0.131	0.23	0.414	0.098	0.40
	**Estradiol **	0.286	0.138	0.12	0.450	0.105	0.63
	**FSH**	0.452	0.135	0.73	0.497	0.130	0.97
	**LH/FSH **	0.643	0.164	0.31	0.464	0.095	0.72
*t* test was used for all variables except for # which was Mann-Whitney test. OHSS: Ovarian hyperstimulation syndrome, AUC: Area under the curve, LH: Luteinizing hormone, TSH: Thyroid stimulating hormone, AMH: Anti-Mullerian hormone, FSH: Follicle-stimulating hormone							

## 4. Discussion

According to the main findings of the current study, although clinical and chemical pregnancy rates were numerically higher in women with OHSS, the pointed difference was not statistically significant in adjusting baseline characteristics. Also, none of the baseline sexual hormones could predict the successful chemical or clinical pregnancy in PCOS women with and without OHSS. Women who suffer from OHSS following IVF protocol may experience significantly lower clinical or chemical pregnancies despite increasing oocyte production and retrieving. It should be noted that applying novel fertility inducible techniques could increase the chance of fertility except in more severe cases. There are conflicting results of the substance's effect on IVF outcomes. As Jiang and colleagues stated, “OHSS, which occurs in the luteal phase or early pregnancy in IVF patients and represents transient abnormal hemodynamics exerts no obvious adverse effects on subsequent pregnancy" (20). However, a study indicated that OHSS in the early course of IVF pregnancies was associated with an increased risk of placental abruption leading to adverse IVF outcomes (21). As shown previously, women with severe OHSS are exposed to more adverse pregnancy outcomes even in the early stages of IVF, such as hemodynamic instability, hypoxia, increasing endogenous estrogens, and increasing secretion of some prostaglandins (22). However, it remains unclear whether the appearance of OHSS in the field of PCOS may affect pregnancy success following IVF. Interestingly, even the onset of this syndrome may be associated with an increased risk of pregnancy. As shown in the present study, both clinical and chemical pregnancies as the consequences of IVF following PCOS may be significantly increased in OHSS women; however, using the multivariable regression modeling and adjusting baseline probable confounders, the effect of OHSS on pregnancy rate disappeared. In other words, the increase in pregnancy rate may have other underlying variables, especially the level of primary sex hormones in cases with this complication.

In the next step, by using ROC curve analysis, we ruled out the role of sex hormones on the pregnancy outcome following IVF. At the same time, a history of amenorrhea and obesity may be the main determinants of fertility failure in such women. In other words, it seems that the role of these hormones, especially AMH, may at least be completely questioned in our community. Systematically reviewing the literatures could show a probable role for serum AMH levels in predicting IVF outcomes. In a review article that examined 32 studies, researchers stated that serum AMH levels were associated with cumulative live birth rates after IVF/intracytoplasmic sperm injection, but no discriminative threshold could be established, so low serum AMH levels should not be should be used as the sole criterion for rejecting IVF treatment, especially in young patients (23). However, some authors have shown that AMH is a reliable parameter in predicting ovarian response to controlled ovarian stimulation as well as the ultrasonographic findings of antral follicle count (24). Some authors even showed that the value of AMH may be more efficient in predicting ovarian response and OHSS than female age and BMI (25). Therefore, it is not yet clear which clinical, or laboratory markers can predict the outcome of IVF in such women and will require further evaluation with a larger sample size.

The study, however, had some limitations. First, the effect of various IVF techniques on pregnancy outcomes in patients in the presence of OHSS was not investigated. Second, the role of OHSS intensity on pregnancy induction results were also not evaluated.

## 5. Conclusion

It can finally be concluded that no significant difference is expected in IVF-related outcomes, including clinical or chemical pregnancy, between the PCOS groups with and without OHSS. In other words, the occurrence of OHSS in such women may not be a main determinant for IVF poorer outcomes. Contrary to popular belief, laboratory markers, especially sex steroids, may not predict the outcome of IVF in these women.

##  Conflict of Interest 

The authors declare that there is no conflict of interest.
